# Poverty and youth disability in China: Results from a large, nationwide, population-based survey

**DOI:** 10.1371/journal.pone.0215851

**Published:** 2019-04-25

**Authors:** Chao Guo, Yanan Luo, Xiaoxue Tang, Ruoxi Ding, Xinming Song, Xiaoying Zheng

**Affiliations:** 1 Institute of Population Research, Peking University, Beijing, China; 2 APEC Health Science Academy (HeSAY), Peking University, Beijing, China; 3 Accommodation Service Center, Peking University, Beijing, China; University of Texas Medical Branch at Galveston, UNITED STATES

## Abstract

**Background:**

Youth with disability contribute to a high burden of disease but are often neglected. This study aims to estimate the prevalence of disability and its association with poverty among Chinese youth aged 15–24 years.

**Methods:**

Data were obtained from a nationally representative population-based cross-sectional survey in 2006 and its follow-up investigations from 2007 to 2013 in 31 provinces of mainland China. A total of 357 856 non-institutionalized youth at age of 15–24 years were investigated. Population weighted numbers and prevalence rates with 95% CI of various types and causes of disabilities for the overall youth were estimated where appropriate. Univariate and multivariate logistic regressions were used to identify the association between poverty and each type of and cause-specific disability.

**Results:**

A weighted number of 3 633 838 youth were living with disability in China, with a prevalence rate of 19.7 per thousand Chinese youth. Youth living in poor households were 3.84 times more likely to be with disability than those living in affluent households (95% CI: 3.56–4.14). Associations were similar for most types of and cause-specific disabilities. Among youth with disability, those from poor households had less healthcare service use (OR: 0.71, 95% CI: 0.61–0.82) than those from affluent households.

**Conclusion:**

A significant number of Chinese youth were living with disability, and poverty is significant associated with the disability among youth. Investment in health and disability prevention are essential to the development of youth, as well as their families and communities.

## Introduction

Disability is becoming an important concern for both developed and developing countries. The World Health Organization (WHO) estimates that more than 650 million people worldwide are living with some form of disability [1]. Disability is a development issue,^1^ which constitutes a sustained burden to both the society and family, especially when a disability occurred in the early stage of life [2]. Due to in a transition from childhood into adulthood, youth who experience disabilities may suffer from severer and longer health and social problems [4], but the disabilities in youth are often overlooked [3]. In 2014, the United Nations Fund for Population Activities (UNFPA) indicated that 1.8 billion youth are the shapers and leaders of our global future, but too many of them continue to grapple with poverty, inequality and health problems [5]. Thus, the UNFPA called for investing in the youth’s health and drew the world’s great attention on the development of youth.

Disability has a bidirectional link with poverty. On the one side, poverty may lead to the onset of disability. Poverty is related to malnutrition, violence and environment pollution exposure, limited access to medical and social sources, and shortage of adequate sustenance and sanitation [6]. Thus, poverty brings high burden on youth’s health outcomes and leads to disability. According to previous evidences, almost 20% of the impoverished population suffer from disability in developing countries [7]. On the other side, people with disability are more likely to experience economic disadvantage [8]. Persons with disability are twice as likely as people without disability to be living in poverty [9].

Although a growing number of studies focus on disability and poverty among adults, literature about disability and poverty on adolescents or youth is very limited. Youth with disability are among the most marginalized and poorest of the world’s youth [10], and poverty of youth is a big challenge to their health investment [5]. The issues of poverty and disability among youth should be even greater concerns for both policymakers and researchers. Compared with older adults, youth often face more stresses from the expectations of them to complete their education, find a regular job, and establish their household and family [11]. Once they fall into poverty, they will face more challenges of health than adults [5], especially for the worst health condition—disability.

In 2016, the total size of youth—defined by the United Nations as the age cohort 15–24 years—is estimated to be 176 billion in China, or 13 percent of the total Chinese population [12]. However, limited studies exist on the relationship between poverty and youth disability. More knowledge about poverty and disability is needed for this population group in China. This study aims to estimate the prevalence of youth with various disabilities, identify the association between poverty and disabilities, as well as the healthcare service use among youth with disability, and explore the trend of poverty among Chinese youth based on nationally representative data. The study results will inform the related policymaking on health promotion in China and contribute to the literature on youth disability and poverty.

## Methods

### Data and ethics statement

Data were obtained from the second China National Sample Survey on Disability (CNSSD) in 2006 and the consecutive follow-up investigations once a year from 2007 to 2013. All data were provided to the authors in an anonymized format. The surveys were approved by the State Council of China (No. 20051104) and conducted within the legal framework governed by the Statistical Law of the People’s Republic of China (1996 Amendment) [13]. The survey protocol and questions were reviewed by experts from the National Bureau of Statistics of China, the China Federation of Persons with Disability, and the Division of Statistics of the United Nations. All survey respondents provided their written consent to participate to this survey. Parental consent for all participants under age 18 were obtained as well.

### Study samples

The 2006 survey focused on the non-institutional population in mainland China and aimed to investigate the prevalence, causes, and severity of disabilities. More than 20 000 interviewers, 50 000 survey assistants, as well as 6 000 doctors of various specialties were involved in the survey. As detailed in our previous work [14], multistage stratified random cluster sampling with probability proportional to size was used to get a nationally representative sample, following standard procedures for complex samples [15]. As a result, 2 526 145 persons in 771 797 households were investigated from a total of 5 964 sites (areas or communities), 2 980 towns (townships or streets), 734 counties (cities or districts) and 31 provinces (autonomous regions or municipalities), approximating 1.93 per 1 000 non-institutionalized residents of China. Among them, there were 7 000 individuals with disability among a total of 357 856 youth aged 15–24 years.

**Fig 1:**
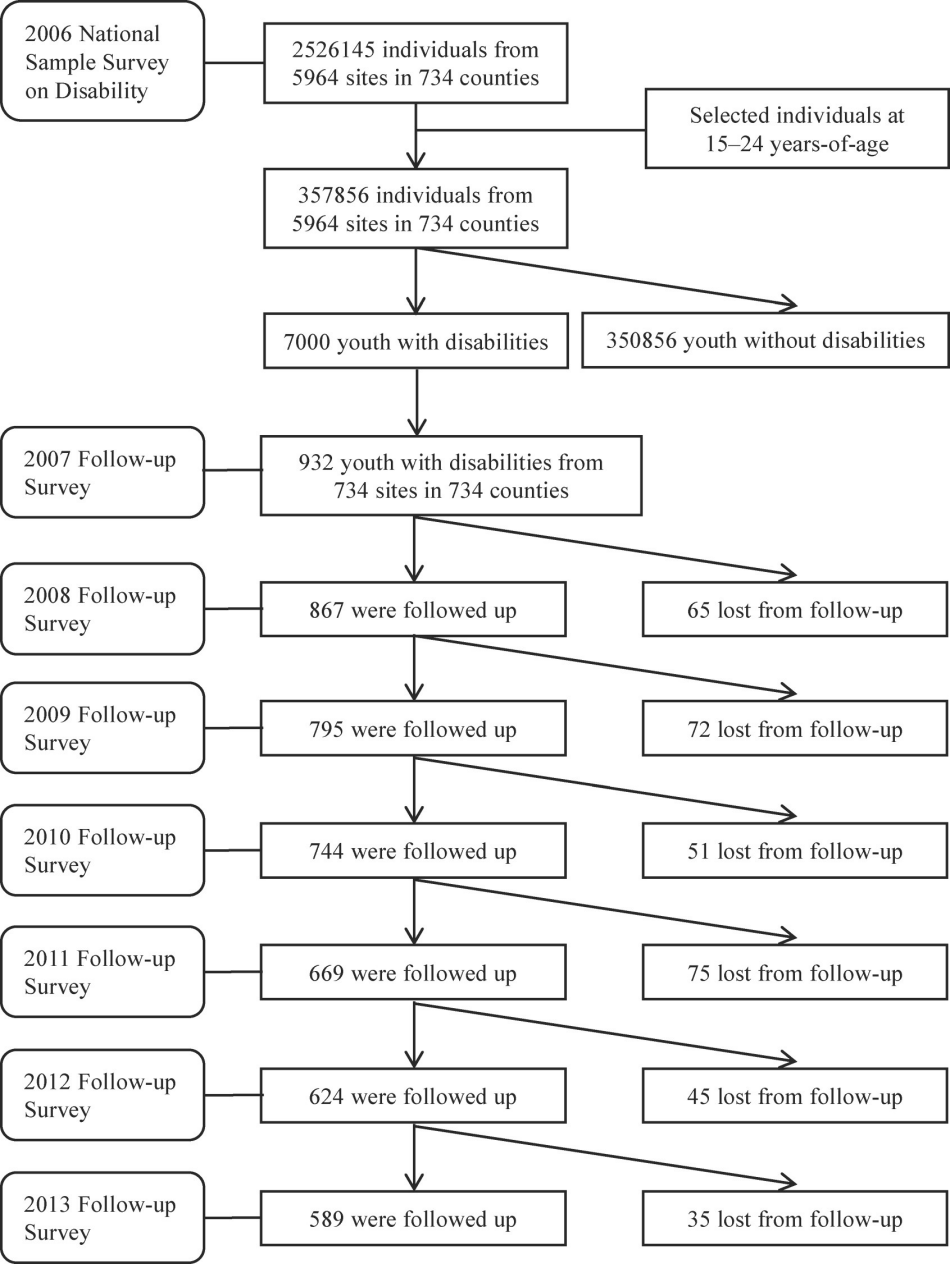
Flowchart of the study samples.

To monitor the living conditions, healthcare services use, as well as the home and community environment of people with disabilities, a sub-sample of people with disability was selected for annual follow-up surveys since 2007. In 2007, the follow-up samples included all diagnosed individuals with disability from 734 study sites which were randomly selected from the 734 counties in 2006, one site for each county. The size of youth with disability aged 15–24 years that was followed up was 932, 861, 795, 744, 669, 624 and 589, respectively, from 2007 to 2013. The reason of lost to follow-up included disappearance, migration and death. The average participation rate of the follow-up studies was 92.6%. A flowchart of study samples was shown in Fig 1.

### Measures

#### Youth

The study population in this study were Chinese youth, referring to all those between the ages of 15 and 24, as defined by the United Nations.

#### Disability

During the survey, each family member of the selected households was interviewed. After collecting the basic information of the households, trained field interviewers used a structured questionnaire according to the ‘Guidelines and Principles for the Development of Disability Statistics’, recommended by the United Nations [16] to inquire about various types of disabilities. Those who responded “yes” to any of the corresponding questions were referred to different designated physicians for further disability screening and confirmation. Designated physicians performed medical examinations and followed diagnostic manuals to make the final diagnosis, if any, to confirm its primary causes. Respondents with multiple positive answers were examined by multiple specialists. The definition and classification of disabilities were established by the Expert Committee of the CNSSD based on the International Classification of Functioning, Disability, and Health (ICF) [17]. The corresponding survey questions and definitions for different types of disabilities were shown in S1 Table.

#### Causes of disabilities

The primary causes of disabilities were diagnosed by specialist doctors with at least 10 years of clinical experience in province-level hospitals or 5 years of clinical experience in county-level hospitals, trained by strict and unified standard [18]. We further categorized the causes according to the International Classification of Diseases 10th Revision (ICD-10) [19] into “infectious and parasitic diseases”, “neoplasms”, “diseases of the blood and immune mechanism”, “endocrine, nutritional and metabolic diseases”, “mental and behavioral disorders”, “diseases of the nervous system”, “diseases of the eye and adnexa”, “diseases of the ear and mastoid process”, “diseases of the circulatory system”, “diseases of the musculoskeletal system”, “being injury when they were in pregnancy, childbirth and the puerperium”, “certain conditions originating in the perinatal period”, “congenital malformations, deformations and chromosomal abnormalities”, “symptoms, signs and findings not elsewhere classified”, “injury, poisoning and external causes”, and others or unknown reasons.

#### Household economic status

Household economic status was indicated by the variable “annual household income per capita”. In each year, it was set as continuous, and further categorized into top, middle and bottom by tertiles of the annual household income per capita to indicate the *affluent*, *medium and poor* households, respectively.

#### Other demographic and socio-economic variables

Age at the time of the survey was set as continuous. Survey respondents were also categorized by gender (male or female), residence (rural areas or urban areas), province region (east, central, west), nationality (Han or minority), education status (educated or illiterate), employment status (employed or not), and family history of disabilities (yes or no).

### Statistical analyses

In 2006 survey, allowing for the complex sampling design, we constructed sample weights using standard weighting procedures calculating the inverse probability of inclusion for an individual survey respondent in the multistage sampling frame [15]. Population weighted numbers and prevalence rates of various types and cause-specific disabilities, with 95% confidence intervals (CIs), for the overall youth were estimated where appropriate. Bonferroni test was used for the difference between household economic statuses. Taylor series linearization method was used to estimate the standard errors of proportions for cross-tabulation tables allowing for first-stage cluster and stratum variance and corresponding 95% CI. Univariate and multivariate logistic regression analyses were used to calculate the odd ratios (ORs) and 95% CI of the association between household economic status and each type and cause-specific disability, as well as the association between poverty and healthcare service use among youth with disability. The mean and standard error of the annual household income per capita over year (2007 to 2013) were calculated with the inflation adjusted. The SAS version. 9.1 (Cary, NC: SAS Institute Inc) was used to perform data analyses. A two-sided P-value less than 0.05 was set as statistically significant.

## Results

### Study population

The study population comprised 357 856 non-institutionalized youth aged 15–24 years, equivalent to a weighted total of 184 855 916. Among them, 51.1% were male; 11.4% were national minority; 72.31% were rural residents; those lived in the east region, central region and west region of China accounted for 40.5%, 32.7% and 26.8%, respectively. Among the youth participants, 82% were with an education level of middle school or high school, and 48.2% were employed currently. For the family information, 33.3%, 35.8% and 30.9% participants were from households with the affluent, median and poor household economic status, respectively; 79.9% were with a family history of disability.

### Poverty and disabilities

In the survey 7 000 youth samples were diagnosed having at least one type of disability. It’s estimated that a weighted number of 3 633 838 youth were living with disability in China. The weighted prevalence rate of disability overall was 19.7 (95% CI: 19.1–20.2) per thousand Chinese youth aged 15 to 24 years. Intellectual disability is the most epidemic disability among youth (8.4 per thousand youth, 95% CI: 8.1–8.8), followed by physical disability (6.8, 6.5–7.1). The sample number, weighted number, and weighted prevalence rate of each type of disability were shown in Table 1.

**Table 1:** The estimated number of Chinese youth with disability and weighted prevalence of disability, by household economic status.

Type of disability	Youth with disability (thousands)	Weighted prevalence, per thousand persons (95% CI)	*P*_*adjust*_ value^a^
Total disability			
Total youth	3 634	19.7 (19.1–20.2)	
Affluent	562	9.1 (8.5–9.7)	<0.001
Medium	1 234	18.6 (17.8–19.5)	
Poor	1 838	32.1 (30.9–33.3)	
Visual disability			
Total youth	221	1.2 (1.1–1.3)	
Affluent	35	0.6 (0.4–0.8)	<0.001
Medium	74	1.1 (0.9–1.3)	
Poor	111	1.9 (1.7–2.2)	
Hearing disability			
Total youth	467	2.5 (2.3–2.7)	
Affluent	78	1.3 (1.1–1.5)	<0.001
Medium	160	2.4 (2.1–2.7)	
Poor	229	4 (3.6–4.4)	
Speech disability			
Total youth	838	4.5 (4.3–4.8)	
Affluent	109	1.8 (1.5–2)	<0.001
Medium	284	4.3 (3.9–4.7)	
Poor	445	7.8 (7.2–8.4)	
Physical disability			
Total youth	1 264	6.8 (6.5–7.1)	
Affluent	207	3.4 (3–3.7)	<0.001
Medium	463	7 (6.5–7.5)	
Poor	594	10.4 (9.7–11.1)	
Intellectual disability			
Total youth	1 560	8.4 (8.1–8.8)	
Affluent	221	3.6 (3.2–3.9)	<0.001
Medium	497	7.5 (7–8)	
Poor	842	14.7 (13.9–15.5)	
Mental disability			
Total youth	583	3.2 (2.9–3.4)	
Affluent	77	1.2 (1–1.5)	<0.001
Medium	178	2.7 (2.4–3)	
Poor	328	5.7 (5.2–6.2)	

^a^ *P*_*adjust*_ value is the *P* value adjusted by *Bonferroni* correction to counteract the problem of multiple comparisons among 3 groups.

The prevalence of disability overall was significantly higher in poor households (32.1 per thousand youth, 95% CI: 30.9–33.3) than medium (18.6, 17.8–19.5) and affluent (9.1, 8.5–9.7) households (p<0.001), and this applied to each type of the six disabilities (Table 1).

In multivariable analysis, youth living in poor households were 3.84 times (95% CI: 3.56–4.14) more likely to be with disability than those living in affluent households were (Table 2). For type-specific disability, poor youth were 5.14 times more likely to be with mental disability than affluent youth (95% CI: 4.21–6.27). Associations were similar for visual disability (OR: 3.30, 95% CI: 2.45–4.45), hearing disability (3.03, 2.45–3.73), speech disability (4.67, 3.94–5.53), physical disability (2.94, 2.59–3.34), and intellectual disability (4.87, 4.33–5.48).

**Table 2:** Univariate and multivariate analysis of the association between poverty and disabilities among youth in China.

Dependent variable	Univariate analysis			Multivariate-adjusted analysis^a^		
	OR	95% CI	*P*_*adjust*_ value^b^	OR	95% CI	*P*_*adjust*_ value^b^
***Any disability***						
Affluent	1		<0.001	1		<0.001
Medium	2.06	1.92–2.21		2.13	1.98–2.30	
Poor	3.56	3.33–3.80		3.84	3.56–4.14	
Visual disability						
Affluent	1		<0.001	1		<0.001
Medium	1.91	1.44–2.54		1.96	1.45–2.63	
Poor	3.40	2.61–4.42		3.30	2.45–4.45	
Hearing disability						
Affluent	1		<0.001	1		<0.001
Medium	1.96	1.61–2.38		1.89	1.54–2.33	
Poor	3.36	2.80–4.04		3.03	2.45–3.73	
Speech disability						
Affluent	1		<0.001	1		<0.001
Medium	2.51	2.14–2.95		2.50	2.11–2.96	
Poor	4.62	3.97–5.38		4.67	3.94–5.53	
Physical disability						
Affluent	1		<0.001	1		<0.001
Medium	2.09	1.86–2.35		2.00	1.77–2.26	
Poor	3.09	2.76–3.46		2.94	2.59–3.34	
Intellectual disability						
Affluent	1		<0.001	1		<0.001
Medium	2.10	1.88–2.35		2.28	2.03–2.57	
Poor	3.98	3.58–4.42		4.87	4.33–5.48	
Mental disability						
Affluent	1		<0.001	1		<0.001
Medium	2.20	1.81–2.66		2.34	1.92–2.87	
Poor	4.56	3.82–5.45		5.14	4.21–6.27	

^a^ The age, gender, residence, province region, nationality, employment status, number of family members and family history of disabilities were controlled in the multivariate-adjusted analysis. The independent variable was household economic status (reference = affluent).

^b^ *P*_*adjust*_ value is the *P* value adjusted by *Bonferroni* correction to counteract the problem of multiple comparisons among 3 groups.

### Poverty and cause-specific prevalence of disability

**Fig 2:**
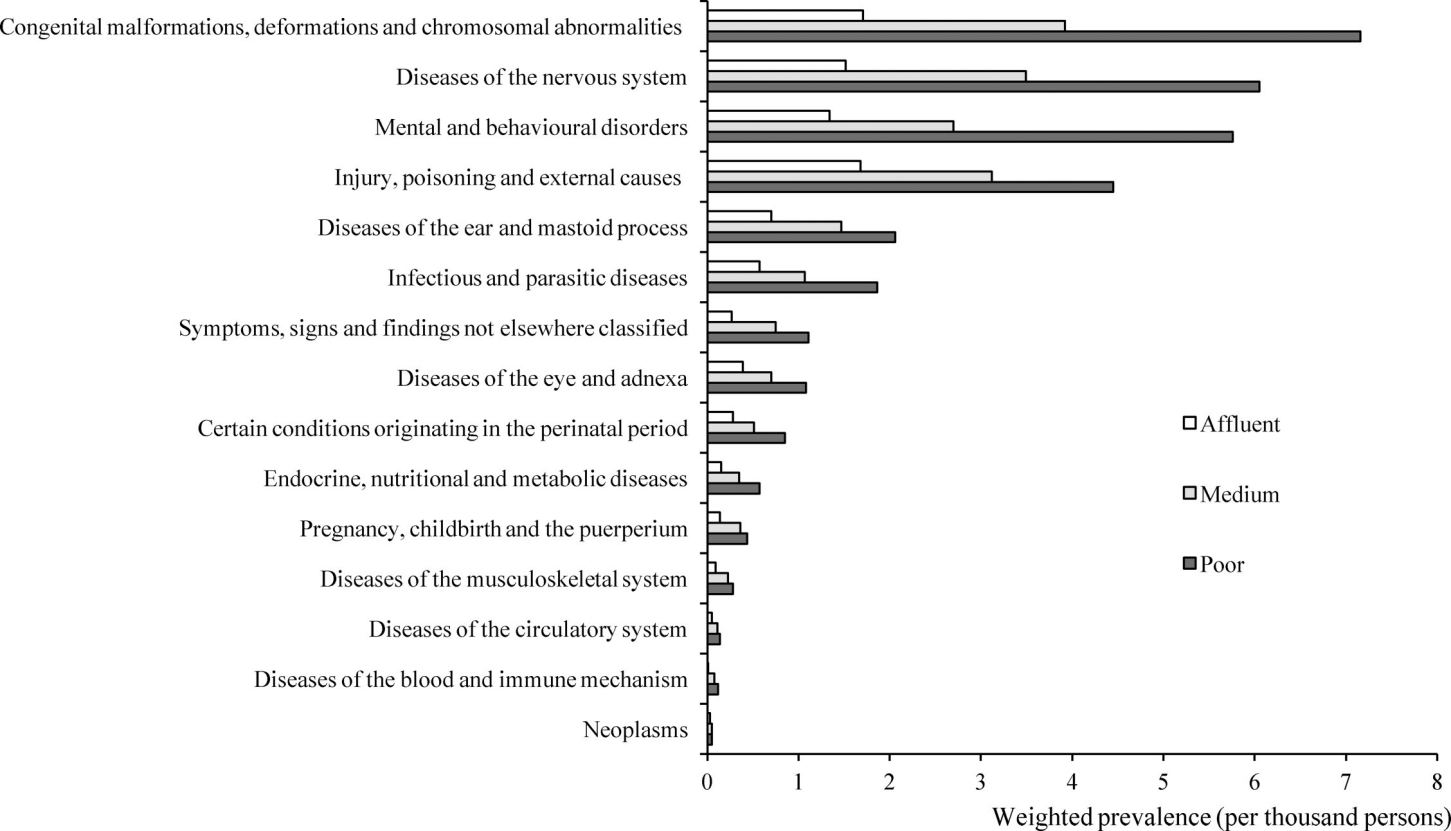
The causes-specific prevalence of disability among Chinese youth, by household economic status.

Congenital malformations, deformations and chromosomal abnormalities was the leading cause (with definite diagnose) of disability among youth, with a weighted number of 773 861 and prevalence of 4.2 per thousand youth. The prevalence of cause-specific prevalence of disability by household economic status was shown in Fig 2. Compared with youth from affluent household, youth living in poor household were found with significant higher prevalence of disabilities caused by most causes. The detailed results of univariate and multivariate analysis of the association between poverty and cause-specific disabilities among youth in China were shown in S2 Table.

### Poverty and healthcare service use among youth with disabilities

In affluent households, 48.8% of youth with disability had ever use healthcare services, compared with 40.3% in medium households and 33.8% in poor households (P<0.001, Table 3). After controlling the sociodemographic variables, in multivariate analysis, youth from poor household were significantly less likely to use curative care use (OR: 0.76, 95% CI: 0.66–0.89), rehabilitation services (0.62, 0.50–0.78), and auxiliary aids use (0.65, 0.48–0.88) compared with those from affluent household.

**Table 3:** Health services use by household economic status among youth with disability in China in 2006.

Health service	Utilization		Univariate analysis			Multivariate-adjusted analysis^a^		
	%	*P*_*adjust*_ value^b^	OR	95% CI	*P*_*adjust*_ value^b^	OR	95% CI	*P*_*adjust*_ value^b^
***Curative care***	34.71	<0.001			<0.001			<0.001
Affluent	42.74		1			1		
Medium	36.42		0.76	0.66–0.89		0.89	0.76–1.03	
Poor	31.03		0.60	0.53–0.69		0.76	0.66–0.89	
***Rehabilitation***	10.68	<0.001			<0.001			<0.001
Affluent	16.61		1			1		
Medium	11.86		0.68	0.55–0.83		0.82	0.67–1.02	
Poor	8.02		0.44	0.36–0.53		0.62	0.50–0.78	
***Auxiliary aids***	5.36	<0.001			<0.001			0.004
Affluent	8.22		1			1		
Medium	6.06		0.72	0.55–0.94		0.89	0.67–1.19	
Poor	3.98		0.46	0.35–0.61		0.65	0.48–0.88	
***Any use***	38.35	<0.001			<0.001			<0.001
Affluent	48.78		1			1		
Medium	40.25		0.70	0.61–0.82		0.84	0.73–0.98	
Poor	33.78		0.54	0.47–0.61		0.71	0.61–0.82	

^a^ The age, gender, residence, province region, nationality, employment status, number of family members and family history of disabilities were controlled in the multivariate-adjusted analysis. The independent variable was household economic status (reference = affluent).

^b^ *P*_*adjust*_ value is the *P* value adjusted by *Bonferroni* correction to counteract the problem of multiple comparisons among 3 groups.

### The household economic status of youth with disabilities over year (2007–2013)

From 2007 to 2013, the annual household income per capita among youth with disabilities was keeping increasing, from less than 5000 RMB in 2007 to over 8000 RMB in 2013. Additionally, the growth rate of annual household income per capita was revving up after 2010 (see S1 Fig).

## Discussion

This study used data from the most recent population–based survey on disability with nationally representative samples in China. The results demonstrate that a significant number of Chinese youth were living with disability and poverty is significantly associated with disability among youth. As reported by United Nations, the global number of adolescents and young adults with disabilities was between 180 and 220 million, which was estimated to be about 16.4 to 20.0 per thousand youth [20]. Our finding showed that the weighted prevalence rate of disability was 19.7 per thousand in Chinese youth, which is consistent with the global level. According to our results, 32.1% of Chinese youth with disability were in poor households. This rate was higher than the rate of developed countries; 10–15% youth with disability aged 5–17 years old were in poor households in the United States [21]. This large discrepancy may partly come from the different definitions of disability, the different study populations, and the differences in economic development levels. Nevertheless, the notable size and prevalence in our finding highlights the importance of disability prevention and rehabilitation among youth in China and other areas with similar settings.

Unsurprisingly, our study found that poverty was associated with the increased likelihood of disability in youth, which was consistent with previous evidences on this relationship in adults [22,23]. Poverty, as one of the most common measures of socioeconomic factors, is also known to have an effect on health of adolescents.^11^ The reasons include the inequalities in health and life expectancy due to social deprivation [24,25], the loss of access to economic and health resources [6,26], and the unfavorable living conditions such as malnutrition, inadequate water and poor sanitation of individuals in poverty. Even in the absence of absolute poverty, relative poverty can lead to stress and social exclusion, which worsens mental and physical health [22,23]. These influences may be more significant for youth because they may face more barriers to finding and maintaining educational, economic, social and cultural opportunities than their older counterparts when suffering from poverty [27], which increases their collective risk of disability.

This study also showed that across various types and causes of disabilities, poverty was highly correlated with increased risk of disabilities in youth. This is similar with previous studies [28–31]. For instance, previous studies indicated that poverty alleviation interventions showed a significant reduction of mental disorders [32], which is consistent with our finding in disabilities caused by mental and behavioural disorders. Although one study in Vietnam reported the negative association between visual impairment and poverty [33], most evidence indicated that poverty was one of the main risk factors of unfavorable conditions for visual impairment [34]. Additionally, socioeconomic inequalities and relative poverty, related to stress and social exclusion, were also found to be associated with physical health and intellectual impairment [29,31].

Furthermore, our results indicated that among youth with disability, those from poor households had the lowest level of health care service utilization. One previous study reported that health care service had a moderate effect on the association between poverty and disability [6]. Improving access to health care for the poor can reduce the rich-poor gap in health [35]. However, in China, the health care utilization among youth with disabilities is at a low level. More than 60% of the youth with disability did not use any kind of health services in China (Table 3). Although local governments may provide medical aid funds and allowances for the persons with disability, the inadequacy of the healthcare system is an appreciable barrier for healthcare use among population with disabilities [36]. Accessible and affordable health care is a particularly important implement preventive measure for youth with disability especially those from the poor households.

Currently, a considerable development agenda, the Poverty Relief Target 2020, has come out in China to alleviate poverty in all its forms. In the past 15 years, China has lifted more than 600 million people out of poverty [37], and this unprecedented achievement was also reflected in the increase of the annual household income per capita in youth with disabilities in our study (see S1 Fig). However, as a nation with the most populous youth, the alleviation of poverty among youth is still an urgent issue for development in China. Given that China is undergoing the rapid social-economic transition and reform of medical system, there is now a window of opportunity for investment in youth’s health.

The current study has several limitations. First, the causality between poverty and youth disability may not be implied directly because our study examines a cross-sectional survey. From this perspective, further prospective studies are encouraged to evaluate how to prevent disability among Chinese youth. Second, due to the shortage of research on poverty and youth disabilities, our study could not be sufficiently compared with other studies from other cultural contexts. Moreover, there may be an underestimate of disability because of possible underreporting if participants did not consider their problems to be serious enough during screening. Regardless of these limitations, our study has estimated the association between poverty and various types of and cause-specific disabilities in China at the national level for the first time.

## Conclusions

In conclusion, our findings indicate that a considerable number of Chinese youth were suffering from disabilities, and poverty was associated with increased risk of various types of and cause-specific disabilities among Chinese youth. The findings may help increase the awareness of youth disability in the general public and for policymakers. Investment in youth’s health, including disability prevention and healthcare improvement, is essential to the achievement of both the Poverty Relief Target 2020 and the Healthy China 2030. This study contributes to the literature on poverty and youth disability in developing nations of a non-Western context as well.

## Supporting information

S1 Fig The household economic status of youth with disabilities, 2007–2013. (TIFF)

S1 Table The corresponding survey questions and definitions for different disability types. (DOCX)

S2 Table Univariate and multivariate analysis of the association between poverty and cause-specific disabilities among youth in China. (DOCX)
