# Supplementary figures and images for: Poverty and youth disability in China: Results from a large, nationwide, population-based survey
Source: PLoS One. 2019 Apr 25;14(4):e0215851. doi: 10.1371/journal.pone.0215851 (PMC6483232; doi:10.1371/journal.pone.0215851)

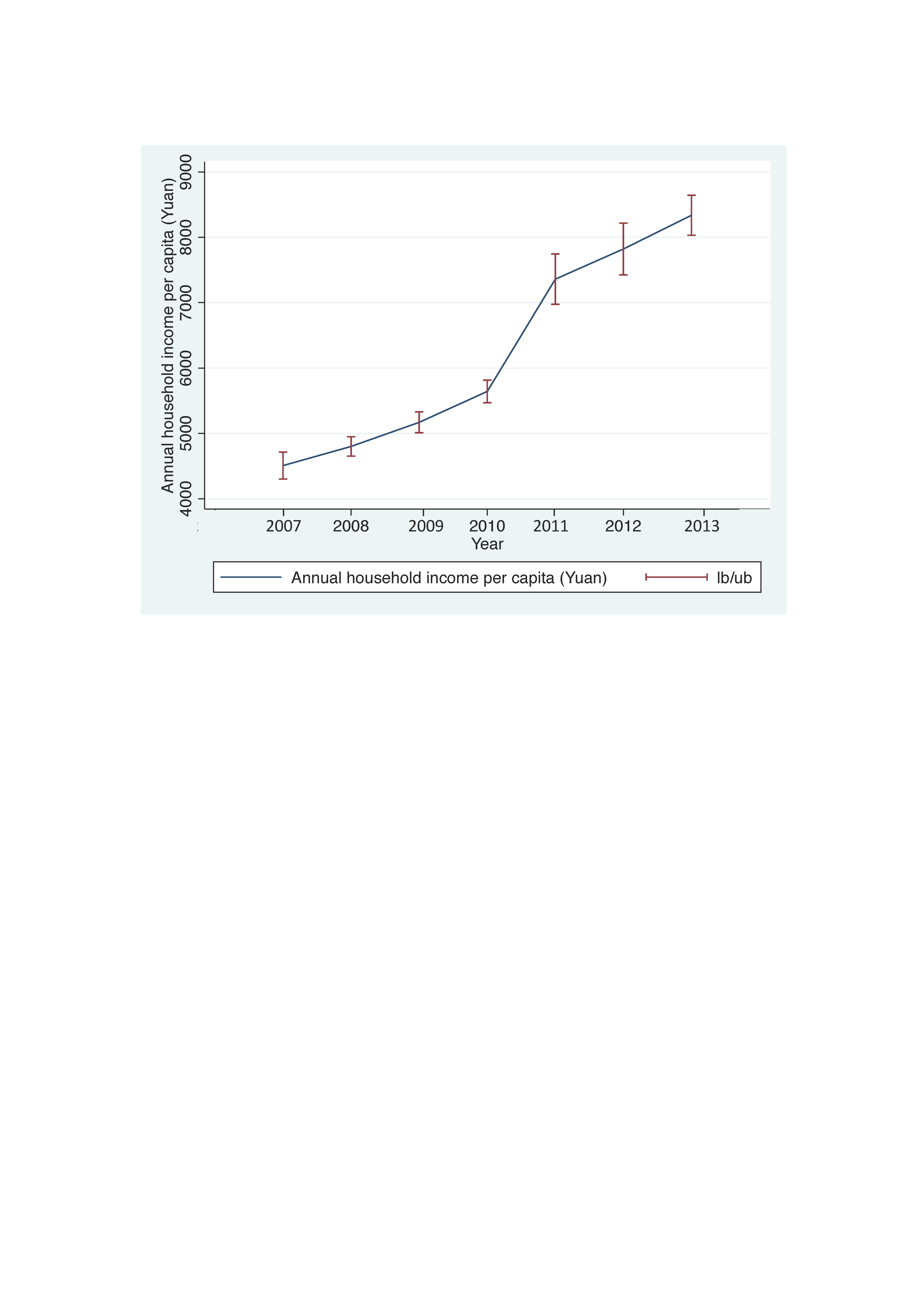

Supplement: S1 Fig — (TIFF) [file pone.0215851.s001.tiff]
