# Supplementary material for: Poverty and youth disability in China: Results from a large, nationwide, population-based survey
Source: PLoS One. 2019 Apr 25;14(4):e0215851. doi: 10.1371/journal.pone.0215851 (PMC6483232; doi:10.1371/journal.pone.0215851)
Supplement: S1 Table — (DOCX) [file pone.0215851.s002.docx]

S1 Table. The corresponding survey questions and definitions for diﬀerent disability types

| Types | Questions in questionnaire | Definition |
| --- | --- | --- |
| Visual disability | Do you or any of your family members has eyesight problem? (Cannot see clearly or cannot see at all)? | Visual disability was defined as poor vision and/or constriction of visual fields (both eyes) from an uncorrectable cause, affecting daily life and social participation. |
| Hearing disability | Do you or any of your family members has hearing problem? (Can he or she answer your question when asking from behind)? | Hearing disability was defined as permanent hearing loss of varying degrees from any cause or the inability to hear at all or to hear clearly any nearby sound or voice, which aﬀect daily life and social activities. |
| Speech disability | Do you or any of your family members has any problem in verbal communication, i.e. cannot speak, speak unclearly and have difficulty in verbal communication? | Speech disability was defined as any type of language disorder. Because successful treatment takes more than 1 year and the disability is generally present for more than 2 years, the patient cannot take part in normal language exchanges, which undermines his or her daily life and participation in social activities. |
| Physical disability | Do you or any of your family members has difficulty in walking, standing, sitting, climbing upstairs, holding, writing, washing or dressing by hand in daily life? | Physical disability was defined as a loss of motor function of varying degrees or to limitations in movements or activities resulting from deformed limbs or body paralysis (palsy) or from deformity caused by damage to the structure or function of those body parts involved in mobility. |
| Intellectual disability | Do you or any of your family members has difficulty in finishing normal learning activities (being late for school, having a poor score, failing to go up to the next grade, or falling out of school midway) or need help because of poor living capability or poor working capability? | Intellectual disability was defined as lower than normal intellectual ability and is accompanied by adaptive behavior disorders. This kind of disability results from impairment of the structure and functions of the nervous system, limits individual activity and participation, and requires all-round, extensive, limited, or intermittent support. |
| Mental disability | Are you or any of your family members forgetful? Do you have difficulty concentrating? Are you unable to control your moods? Are you subject to strange or out-of-the-ordinary behavior? Is there addiction to alcohol or drugs? For those aged 17 years and below, the following questions were asked additionally: Are you/they lack of eye contact, listening but not hearing? Are you/they lonely with narrow interests, and often addicted monotonous and repeated activities? Is there any language or non-language barrier to intercommunication? | Mental disability was defined as any psychiatric condition of >1-year duration, manifesting as a cognitive, affective, or behavioral disorder and affecting the daily life and social participation of the patient. |
