# Supplementary material for: Poverty and youth disability in China: Results from a large, nationwide, population-based survey
Source: PLoS One. 2019 Apr 25;14(4):e0215851. doi: 10.1371/journal.pone.0215851 (PMC6483232; doi:10.1371/journal.pone.0215851)
Supplement: S2 Table — (DOCX) [file pone.0215851.s003.docx]

S2 Table. Univariate and multivariate analysis of the association between poverty and cause-specific disabilities among youth in China

| Household economic status (reference=Affluent) | Univariate analysis | | |  | Multivariate-adjusted analysis^a^ | | |
| --- | --- | --- | --- | --- | --- | --- | --- |
|  | OR | 95% CI | *P* |  | OR | 95% CI | *P* |
| Congenital malformations, deformations and chromosomal abnormalities | | | | |  |  |  |
| Medium | 2.32 | 1.97–2.72 | <0.001 |  | 2.31 | 1.95–2.73 | <0.001 |
| Poor | 4.10 | 3.53–4.77 | <0.001 |  | 4.33 | 3.67–5.12 | <0.001 |
| Diseases of the nervous system | |  |  |  |  |  |  |
| Medium | 2.25 | 1.90–2.67 | <0.001 |  | 2.25 | 1.88–2.69 | <0.001 |
| Poor | 3.85 | 3.28–4.53 | <0.001 |  | 4.05 | 3.39–4.83 | <0.001 |
| Mental and behavioural disorders | |  |  |  |  |  |  |
| Medium | 2.05 | 1.69–2.49 | <0.001 |  | 2.08 | 1.71–2.54 | <0.001 |
| Poor | 4.34 | 3.64–5.18 | <0.001 |  | 4.64 | 3.82–5.63 | <0.001 |
| Injury, poisoning and external causes | |  |  |  |  |  |  |
| Medium | 1.900 | 1.61–2.26 | <0.001 |  | 1.85 | 1.55–2.22 | <0.001 |
| Poor | 2.8 | 2.38–3.29 | <0.001 |  | 2.5 | 2.08–3 | <0.001 |
| Diseases of the ear and mastoid process | |  |  |  |  |  |  |
| Medium | 2.24 | 1.72–2.9 | <0.001 |  | 2.25 | 1.71–2.96 | <0.001 |
| Poor | 3.24 | 2.52–4.17 | <0.001 |  | 3.09 | 2.33–4.09 | <0.001 |
| Infectious and parasitic diseases | |  |  |  |  |  |  |
| Medium | 1.93 | 1.45–2.59 | <0.001 |  | 1.67 | 1.23–2.26 | 0.001 |
| Poor | 3.33 | 2.54–4.38 | <0.001 |  | 2.72 | 2.02–3.68 | <0.001 |
| Diseases of the eye and adnexa | |  |  |  |  |  |  |
| Medium | 2.64 | 1.81–3.86 | <0.001 |  | 2.61 | 1.76–3.88 | <0.001 |
| Poor | 3.98 | 2.77–5.73 | <0.001 |  | 3.94 | 2.64–5.88 | <0.001 |
| Convulsions |  |  |  |  |  |  |  |
| Medium | 1.79 | 1.26–2.54 | 0.001 |  | 1.73 | 1.2–2.5 | 0.004 |
| Poor | 2.89 | 2.08–4.02 | <0.001 |  | 2.4 | 1.66–3.49 | <0.001 |
| Certain conditions originating in the perinatal period | | |  |  |  |  |  |
| Medium | 1.62 | 1.08–2.42 | 0.018 |  | 1.9 | 1.25–2.88 | 0.003 |
| Poor | 2.57 | 1.77–3.75 | <0.001 |  | 3.4 | 2.23–5.18 | <0.001 |
| Endocrine, nutritional and metabolic diseases | | |  |  |  |  |  |
| Medium | 2.14 | 1.24–3.7 | 0.006 |  | 2.03 | 1.15–3.59 | 0.014 |
| Poor | 3.76 | 2.25–6.27 | <0.001 |  | 3.71 | 2.11–6.5 | <0.001 |
| Pregnancy, childbirth and the puerperium | | |  |  |  |  |  |
| Medium | 2.77 | 1.62–4.76 | <0.001 |  | 3.38 | 1.93–5.93 | <0.001 |
| Poor | 3.04 | 1.77–5.21 | <0.001 |  | 4.01 | 2.21–7.28 | <0.001 |
| Diseases of the musculoskeletal system | |  |  |  |  |  |  |
| Medium | 2.35 | 1.23–4,.51 | 0.01 |  | 2.17 | 1.1–4.27 | 0.026 |
| Poor | 3 | 1.59–5.68 | 0.001 |  | 2.72 | 1.36–5.47 | 0.005 |
| Diseases of the circulatory system | |  |  |  |  |  |  |
| Medium | 2.55 | 0.99–6.56 | 0.053 |  | 2.9 | 1.08–7.77 | 0.034 |
| Poor | 2.97 | 1.16–7.6 | 0.023 |  | 3.35 | 1.19–9.47 | 0.022 |
| Diseases of the blood and immune mechanism | | |  |  |  |  |  |
| Medium | 9.17 | 1.16–72.35 | 0.036 |  | 7.35 | 0.9–60.18 | 0.063 |
| Poor | 14.5 | 1.9–110.84 | 0.01 |  | 9.16 | 1.1–76.05 | 0.04 |
| Neoplasms |  |  |  |  |  |  |  |
| Medium | 1.27 | 0.34–4.74 | 0.719 |  | 1.45 | 0.36–5.75 | 0.6 |
| Poor | 1.67 | 0.47–5.93 | 0.425 |  | 2.35 | 0.57–9.65 | 0.236 |
| Others or unknown reasons | |  |  |  |  |  |  |
| Medium | 2.01 | 1.75–2.32 | <0.001 |  | 2 | 1.72–2.3 | <0.001 |
| Poor | 3.85 | 3.38–4.38 | <0.001 |  | 3.96 | 3.43–4.57 | <0.001 |

^a^ The age, gender, residence, province region, nationality, employment status, and family history of disabilities were controlled in the multivariate-adjusted analysis. The independent variable was household economic status (reference=affluent).
